# Supplementary material for: The circadian clock regulates receptor-mediated immune responses to an herbivore-associated molecular pattern
Source: bioRxiv. 2024 Nov 8:2024.11.06.622352. Preprint. [Version 1] doi: 10.1101/2024.11.06.622352 (PMC11581021; doi:10.1101/2024.11.06.622352)
Supplement: Supplement 7 — Figure S1. Clustering analysis of DEGs in response to w + H2O and w + In11. Figure S2. Venn diagrams of DEGs in response to w + H2O applied at different times of the day. Figure S3. Genome-wide distribution and abundance of CBS and EE motifs in cowpea promoters. Figure S4. AtLHY weakly activates the VuKTI promoter in a CBS-dependent manner. [file NIHPP2024.11.06.622352v1-supplement-7.pdf]

bioRxiv preprint doi: https://doi.org/10.1101/2024.11.06.622352; this version posted November 8, 2024. The copyright holder for this preprint (which was not certified by peer review) is the author/funder, who has granted bioRxiv a license to display the preprint in perpetuity. It is made available under aCC-BY 4.0 International license.

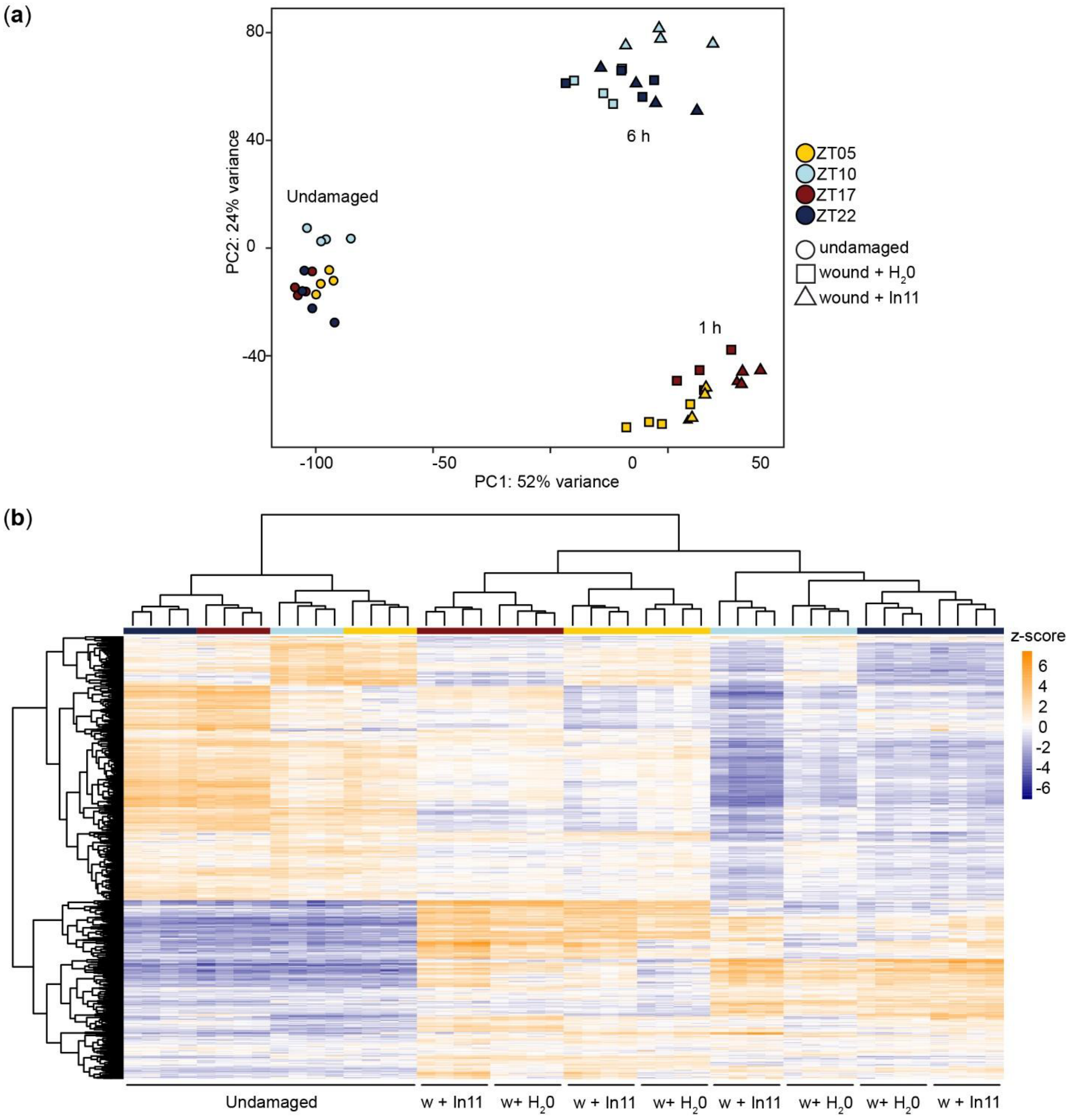

Figure S1. Clustering analysis of DEGs in response to w + H_2O and w + In11. (a) Principal component (PC) analysis of differentially expressed genes (DEGs) across all samples. (b) Hierarchical clustering of samples according to the expression pattern of 847 In11-responsive DEGs across all samples.

bioRxiv preprint doi: https://doi.org/10.1101/2024.11.06.622352; this version posted November 8, 2024. The copyright holder for this preprint (which was not certified by peer review) is the author/funder, who has granted bioRxiv a license to display the preprint in perpetuity. It is made available under aCC-BY 4.0 International license.

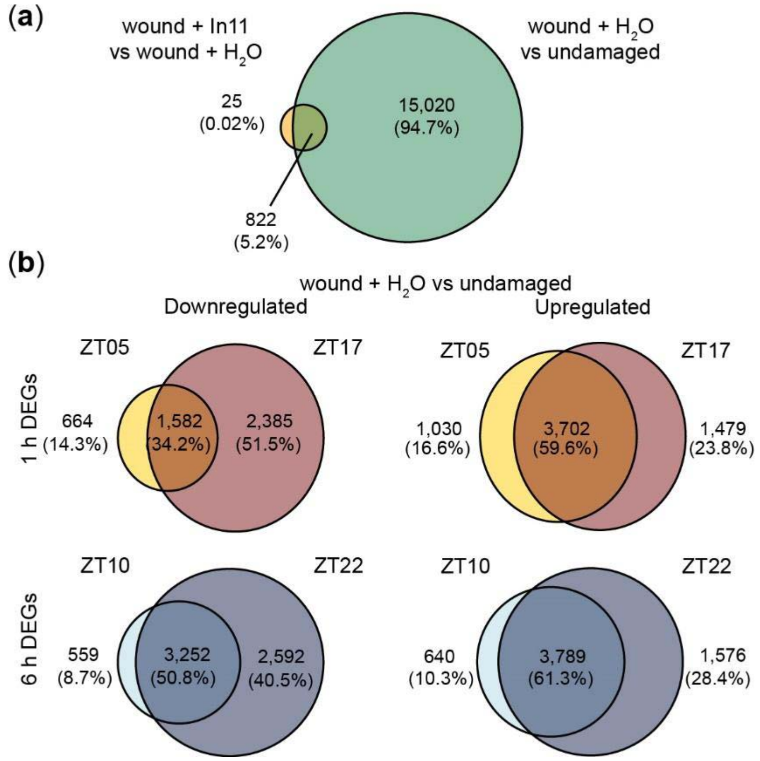

Figure S2. Time -of-day response to wound + H_2O vs undamaged. Venn diagrams indicating the number of shared and unique up and down-regulated genes (a) In11 vs wound across all time points, and (b) 1h (ZT5 vs ZT17) and 6h (ZT10 vs ZT22) after daytime or nighttime wounding (wound + H_2O vs undamaged).

bioRxiv preprint doi: https://doi.org/10.1101/2024.11.06.622352; this version posted November 8, 2024. The copyright holder for this preprint (which was not certified by peer review) is the author/funder, who has granted bioRxiv a license to display the preprint in perpetuity. It is made available under aCC-BY 4.0 International license.

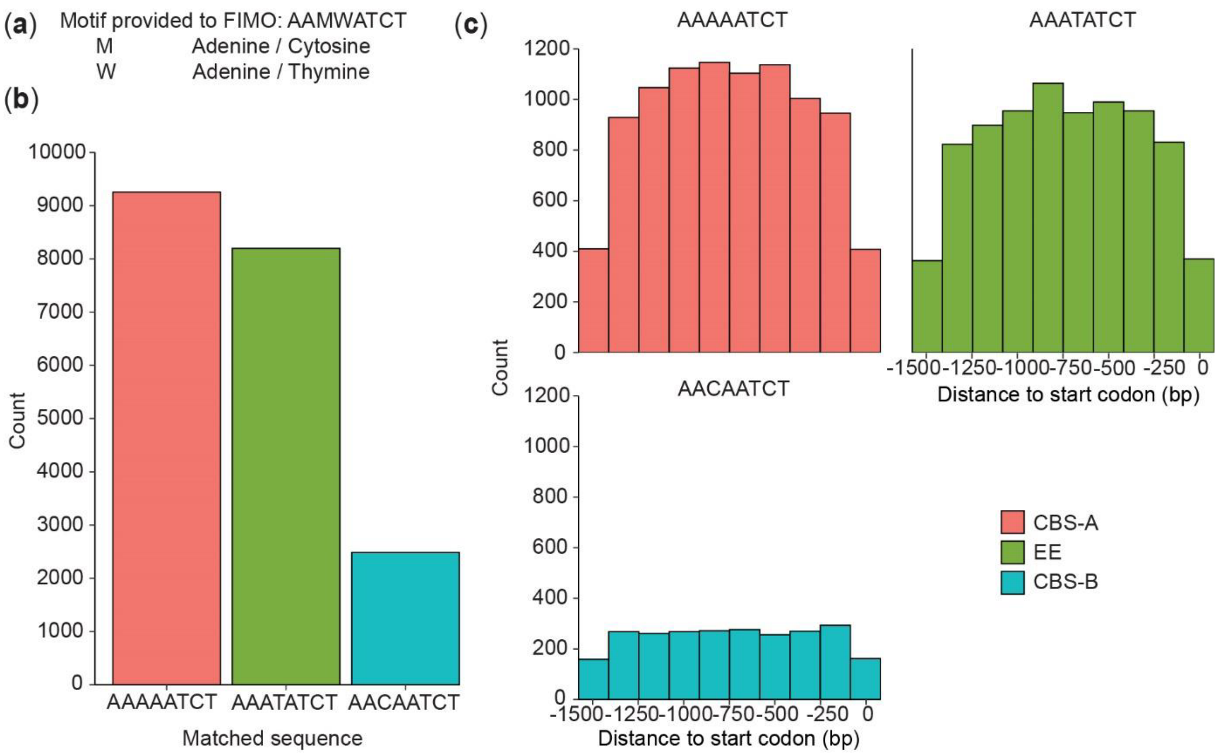

Figure S3. Genome-wide distribution and abundance of CBS and EE motifs in cowpea promoters. Predicted promoter sequences (1.5 kb upstream start codon) were retrieved from the cowpea genome for a circadian clock cis element analysis. (a) Motif provided to Find Individual Motif Occurrences (FIMO) software. (b) Sequence and count for known and novel motifs found in the promoters. CCA1 Binding site A (CBS-A), Evening element (EE), CBS-B, CBS-like. (c) Motif location distribution in the promoters in 250 base pair (bp) bins.

bioRxiv preprint doi: https://doi.org/10.1101/2024.11.06.622352; this version posted November 8, 2024. The copyright holder for this preprint (which was not certified by peer review) is the author/funder, who has granted bioRxiv a license to display the preprint in perpetuity. It is made available under aCC-BY 4.0 International license.

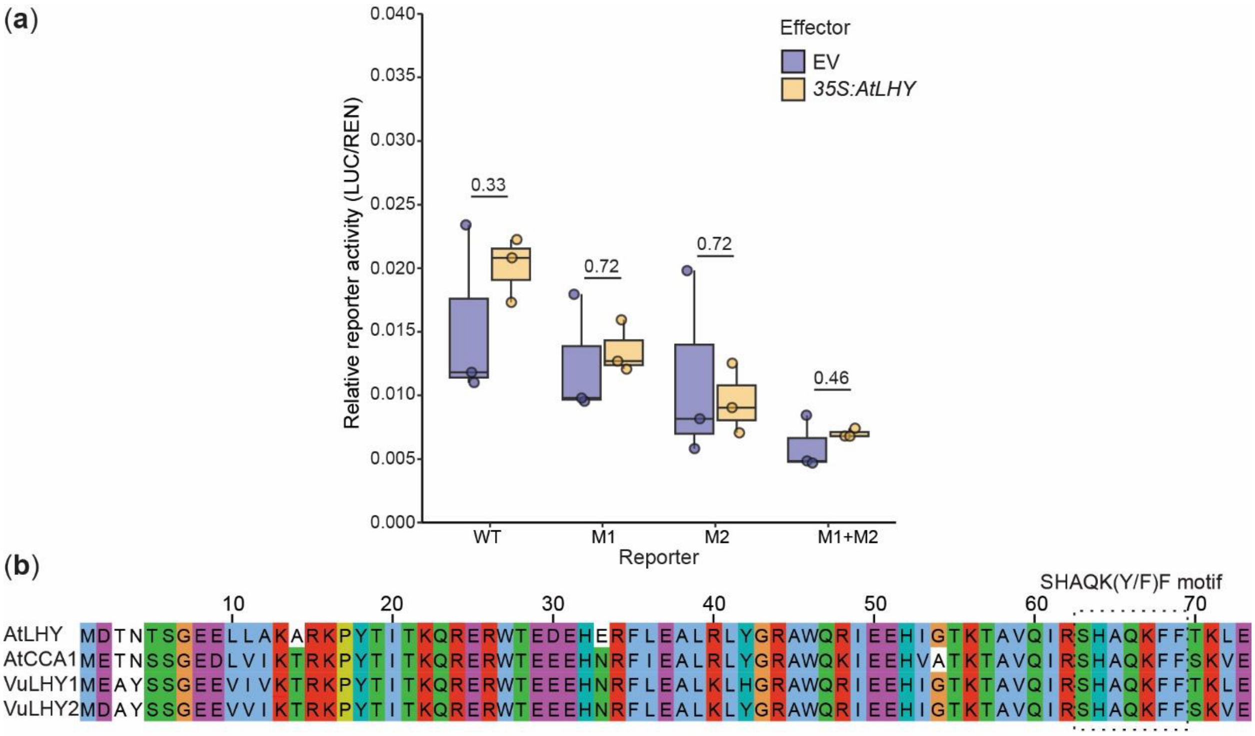

Figure S4. AtLHY weakly activates the *VuKTI* promoter in a CBS-dependent manner. The effect of the AtLHY protein on the activity of the LUC reporters. (a) At 72 h LUC activity was measured with 35S:AtLHY protein co-expressed in a separate agrobacterium strain. WT=CBS, CBS-L, M1 = ΔCBS, CBS-L, M2 = CBS, ΔCBS-L. Relative reporter activity was calculated by normalization against *35S:Renilla*. Reporters final OD_{600}=0.3 and effectors final OD_{600}=0.4 Significant differences in the mean (*) were determined by a two-sided t-test of each effector vs. EV (n = 3, α= 0.05). (b) Alignment of the amino acid sequence of the Myb-like DNA binding domain for AtLHY, AtCCA1, VuLHY1 and VuLHY2. Color scale according to ClustalW.
